# Crossing a Century: Black Male Resilience, Spirituality, and New Horizons

**DOI:** 10.1093/geroni/igaf122.4356

**Published:** 2025-12-31

**Authors:** Stephanie Boddie, Jocelyn McGee, Rebecca Meraz

**Affiliations:** Baylor University, Waco, Texas, United States; Baylor University, Waco, Texas, United States; Baylor University, Waco, Texas, United States

## Abstract

Background and Objective The U.S. centenarian population has nearly tripled over the last three decades (Schaeffer, 2024). Centenarians are disproportionately white women, with only 8% identifying as Black (Schaeffer, 2024). This study explores the lived experiences of two Black men who achieved extreme longevity, highlighting the role of spirituality as a protective factor that fosters resilience and provides insights into navigating stressful life events. Research Method Data were drawn from oral history collections of Black elders. The analysis employed Elder’s (1998) Life Course Theory and Dannefer’s (2003) Cumulative Advantage/Disadvantage (CAD) Theory to conduct thematic analysis of two oral histories. Findings Charles Wiggins (d. 103) and Louis Tucker (d. 105) were both raised in the Black church and sustained strong religious and spiritual practices throughout their lives. Over the course of a century, they endured wars, illness, discrimination, and domestic terrorism. Their spirituality, coupled with resilience cultivated in early life, served as a buffer against stress in later years (Jones et al., 2022). Both men also maintained social engagement, independence, and mobility, including driving, well into advanced age. Discussion and Implications This study underscores the importance of spirituality as a dimension of resilience in extreme longevity, particularly among Black men. Findings suggest that attention to spiritual practices can enhance understanding of health, social support, and family dynamics across the life course. Implications extend to caregiving practices and gerontological research, pointing to the need for greater consideration of cultural and spiritual factors in supporting centenarians.

